# Can we predict discordant RECIST 1.1 evaluations in double read clinical trials?

**DOI:** 10.3389/fonc.2023.1239570

**Published:** 2023-10-04

**Authors:** Hubert Beaumont, Antoine Iannessi

**Affiliations:** Sciences, Median Technologies, Valbonne, France

**Keywords:** clinical trial, Interobserver variation, RECIST, computed tomography, lung cancer

## Abstract

**Background:**

In lung clinical trials with imaging, blinded independent central review with double reads is recommended to reduce evaluation bias and the Response Evaluation Criteria In Solid Tumor (RECIST) is still widely used. We retrospectively analyzed the inter-reader discrepancies rate over time, the risk factors for discrepancies related to baseline evaluations, and the potential of machine learning to predict inter-reader discrepancies.

**Materials and methods:**

We retrospectively analyzed five BICR clinical trials for patients on immunotherapy or targeted therapy for lung cancer. Double reads of 1724 patients involving 17 radiologists were performed using RECIST 1.1. We evaluated the rate of discrepancies over time according to four endpoints: progressive disease declared (PDD), date of progressive disease (DOPD), best overall response (BOR), and date of the first response (DOFR). Risk factors associated with discrepancies were analyzed, two predictive models were evaluated.

**Results:**

At the end of trials, the discrepancy rates between trials were not different. On average, the discrepancy rates were 21.0%, 41.0%, 28.8%, and 48.8% for PDD, DOPD, BOR, and DOFR, respectively. Over time, the discrepancy rate was higher for DOFR than DOPD, and the rates increased as the trial progressed, even after accrual was completed. It was rare for readers to not find any disease, for less than 7% of patients, at least one reader selected non-measurable disease only (NTL). Often the readers selected some of their target lesions (TLs) and NTLs in different organs, with ranges of 36.0-57.9% and 60.5-73.5% of patients, respectively. Rarely (4-8.1%) two readers selected all their TLs in different locations. Significant risk factors were different depending on the endpoint and the trial being considered. Prediction had a poor performance but the positive predictive value was higher than 80%. The best classification was obtained with BOR.

**Conclusion:**

Predicting discordance rates necessitates having knowledge of patient accrual, patient survival, and the probability of discordances over time. In lung cancer trials, although risk factors for inter-reader discrepancies are known, they are weakly significant, the ability to predict discrepancies from baseline data is limited. To boost prediction accuracy, it would be necessary to enhance baseline-derived features or create new ones, considering other risk factors and looking into optimal reader associations.

## Highlights

In RECIST BICR trials with double reads there is large variability in tumor measurement and localization.

Discrepancy rates can be modeled over time.

Few discrepancies can be predicted from baseline evaluations.

## 1 Introduction

In 2004, the Food and Drug Administration recommended double radiology reads for clinical trials with blinded independent central review (BICR) to minimize evaluation bias (1). Due to the variabilities in observers’ evaluations, diagnostic results can be discordant (2). In such situations, a third radiologist, the “adjudicator”, is required so that a final decision can be made (3). The rate of discordance (a.k.a. the adjudication rate), which is the number of discordant evaluations out of the total number of patients in the trial, is the preferred high-level indicator that summarizes the overall reliability of assessments of trials (4). The monitoring of observers’ variability through the adjudication rate requires a burdensome process, which all stakeholders aim to make cost-effective (5, 6), with the goal of minimizing inter-reader discordances.

The Response Evaluation Criteria In Solid Tumor (RECIST) (7) is widely used and accepted by regulatory authorities for evaluating the efficacy of oncology therapies in clinical trials with imaging. The very purpose of RECIST is to assign each patient to one of the classes of response to therapy: progressive disease (PD), stable disease (SD) or responders (partial or complete response [PR, CR]) (7). When categorized as PD, patients must be withdrawn from the study and their treatment stopped. Depending on the development phase of the drug (8), different trial endpoints can be derived from the RECIST assessments. Indeed, in phase 2, the study endpoint generally relates to response (responder vs non-responder) while in phase 3, it relates to progression. Each of these trial endpoints have their own statistical features linked to their respective kind of inter-reader discrepancy (KoD). Therefore, for a given trial, the most relevant KoD to monitor can differ from another trial.

During trials, patients undergo a sequential radiological RECIST 1.1 evaluation with a probability of discrepancy occurring at each radiological timepoint response (RTPR). We can hypothesize that each of the KoDs has a different likelihood of occurrence over time. We can also assume that “at-risk-periods” and “at-risk-factors” exist for discrepancies occurring during patient follow-up. From an operational standpoint, confirming these assumptions would be particularly relevant for BICR trials with double reads (3, 9).

Clinical trials can often take a long time to complete, during which changes may arise from various sources: readers may become more experienced, tumor shapes may complexify, or operational parameters may have unintended impacts. Thus, it is essential to assess the broad trends while the trial is in progress to gain insight into any potential changes that may have occurred and their effects on the trial’s ultimate results.

The issue around RECIST subjectivity has been widely discussed (10, 11) and there is consensus on risk factors related to disease evaluation at baseline. These risk factors can be grouped into tumor selection (12) and quantification (13). However, it is still unclear which of these risk factors most impact the response and how they interact with each other to allow prediction at baseline as to which reads are more likely to become discrepant at follow-up. In addition, a data-driven approach using machine learning (ML) could be an opportunity to test whether baseline-derived features are predictors of inter-reader discordances.

We conducted a retrospective analysis of inter-reader discrepancies in five BICR RECIST lung trials with double reads with three primary objectives: 1) to investigate the discrepancy rate over time, aiming to identify influential high-level factors, 2) to identify risk factors for discrepancies related to RECIST-derived features at baseline, and 3) to use confirmed risk factors for discrepancies to evaluate predictive models.

## 2 Methods

### 2.1 Study data inclusion criteria

Our retrospective analysis included results from five BICR clinical trials (Trials 1-5) that evaluated immunotherapy or targeted therapy for lung cancer (**Table 1**). The selected BICR trials were conducted between 2017 and 2021 with double reads with adjudication based on RECIST 1.1 guidelines. All data were fully blinded regarding study sponsor, study protocol number, therapeutic agent, subject demographics, and randomization. For these five trials, a total of 1724 patients were expected, involving 17 radiologists. The central reads were all performed using the same radiological reading platform (iSee; Median Technologies, France).

**Table 1:** Description of included trials.

Trial ID	Phase	Ranked adjudicated endpoints	Number of patients and visits	Visit period (weeks)	Average number of visits per patient	Per patient, mean and median follow-up duration (days)
**Trial 1**	3	DOPD, BOR, DOFR	nPatient=333 nTP=2054	6 then 12 after 54 weeks	6	mean: 226 [212,240] median: 192
**Trial 2**	3	DOPD, BOR	nPatient=493 nTP=6006	6 then 12 after 48 weeks	12	mean: 234 [222; 246] median: 217
**Trial 3**	2	BOR, DOFR, DOPD	nPatient=243 nTP=5260	8 weeks	14	mean: 514 [456; 571] median: 315
**Trial 4**	3	DOPD	nPatient=276 nTP=2796	8 then 12 after cycle 19	19	mean: 516 [479; 553] median: 506
**Trial 5**	3	DOPD	nPatient=379 nTP=2554	6 then 9 after 48 weeks	6	mean: 248 [233; 264] median: 198

In all trials, the indication was locally advanced (>III-B) or metastatic non-small cell lung cancer treated with immunotherapy evaluated with RECIST 1.1. Trials 1 and 5 related to first line treatment, and all trials included a control group except Trial 3. For Trials 1 and 2, measurable disease was an inclusion criterion at baseline and brain lesions were not excluded (selected as NTLs). Adjudication endpoints were DOPD, BOR, and DOFR.

BOR, best overall rate; DOFR, date of first response; DOPD, date of progressive disease; NTL, non-target lesion.

### 2.2 Read paradigm

Two independent radiologists performed the review of each image and determined the RTPR in accordance with RECIST 1.1. According to the trial’s endpoints (response or progression), specific KoDs triggered adjudications that were pre-defined in an imaging review charter. The adjudicator reviewed the response assessments from the two primary readers and endorsed the outcome of one of the readers, providing rationale to endorse the adjudicator’s assessment.

### 2.3 Analysis plan

We considered four KoDs related to the standard endpoints used on trials:

A. Two related to event or time-to-event of disease progression:
1. Progressive disease declared (PDD): Discrepant PD detection when only one of the readers declared PD during the follow-up.
2. Date of progressive disease (DOPD): Discrepant dates of PD detection as either one reader did not detect PD at all during the follow-up or both readers declared a PD but at different dates.
B. Two related to event or time-to-event of disease response:
3. Best overall response (BOR): Discrepant reporting of the best among all overall responses (CR was best, followed by PR, SD, and then PD) during follow-up. To simplify the analysis, we adopted the definition of BOR from the RECIST group (7) but without response confirmation and minimal SD duration definition. This definition is also known as best time point response.
4. Date of first response (DOFR): Discrepant CR or PR of detection date as either one of the readers declared no CR or PR during the follow-up or both readers detected a first CR or PR but at different dates.

One example of patient follow-up with corresponding KoDs is provided in **Table 2**.

**Table 2:** Example of KoDs.

Evaluation	Timepoints Evaluation						Kind of Discrepancy			
	TP1	TP2	TP3	TP4	TP5	TP6	BOR	DOFR	PPD	DOPD
*Reader 1*	SD	PR	PR	CR	PD	PD	*CR*	*TP2*	*YES*	*TP5*
*Reader 2*	SD	SD	PR	PR	PR	PR	*PR*	*TP3*	*NO*	*NA*

During double read evaluations by Reader 1 and Reader 2 over six time points, the discrepant values of the four KoDs were reported in the rightmost columns.

BOR, best overall response; CR, complete response; DOFR, date of first response; DOPD, declaration of progression of disease; NA, not applicable; PD, progressive disease; PPD, progressive disease declared; PR, partial response; SD, stable disease; TP, time point.

For each trial and KoD, our study addressed the distribution of the discrepancies, the risk factors for discrepancies, and their predictions:

#### 2.3.1 Distribution of discrepancies

a) The rate of discrepant patients

At trial completion (or near completion for Trial 1), we measured the ratio of the number of patients for whom a KoD was detected during their follow-up to the number of patients.

b) The rate of discrepant patients over time

The temporal discrepancy rate can be written as the cumulative function of the distribution of probability for discrepancy at each time point *PDisc(t)* multiplied by the survival curve *Surv(t)* (or drop out curve) and convolved by the function of the number of patients included at each time point *PatIncl(t)* (Equation 1).


Equation 1 $$RateOfDisc\left(t\right)=\int_{t=0}^{t}PatIncl\left(\tau \right)*\left(PDisc\left(\tau \right)\cdot Surv\left(\tau \right)\right)d\tau$$

For each KoD and for each trial, we analyzed the rate of discrepancies over time. This rate can be simply calculated as the ratio of the number of discrepant patients included from the beginning of the study to the number of patients included from the start of the study during the same period (Equation 2).


Equation 2 $$RateOfDisc\left(t\right)=\frac{\sum_{t=0}^{t}Numb\;of\;discrepant\;pat\left(t\right)}{\sum_{t=0}^{t}Number\;of\;included\;Pat\left(t\right)}$$

Where *t* is the time, *t=0* at trial onset (first patient in).

c) The proportion of discrepancies occurring at each follow-up time

For each KoD and each trial, we computed the average proportion of discrepant patients occurring between two time points (Equation 3). These proportions, which are functions of the progression free survival (PFS) curve (therefore of the survival curve) and the probability of KoD occurrence during an interval of time, are displayed along with the proportions of patients still evaluated until this time point.


Equation 3 $$PropDisc\left(FU\right)=100*\frac{\sum_{t=FU-1}^{t=FU}Numb\;of\;discrepant\;pat\left(t\right)}{\sum_{t=Baseline}^{t=FUmax}Number\;of\;discrepant\;pat\left(t\right)}$$

Where *FU* is an interval in patient follow-up time and *t* ranging [*Baseline; FUmax*] is the maximum follow-up duration measured in the study.

d) The probability of discrepancy during follow-up

For each KoD and each trial, as presented earlier in Equation 1, we provided the probability of a patient having a discrepant diagnosis during a given follow-up interval. We computed this probability as the ratio of the number of discrepant diagnoses to the number of patients that were evaluated during this follow-up interval of time (Equation 4).


Equation 4 $$PDisc\left(FU\right)=\frac{\sum_{t=FU-1}^{t=FU}Number\;of\;discrepant\;pat\left(t\right)}{\sum_{t=FU-1}^{t=FU}Number\;of\;pat\;\left(t\right)}$$

Where *FU* is a given follow-up time point.

Our discrepancy analysis considered only patients who underwent at least one follow-up visit after baseline, for a clearer display, we resampled curves in a standardized time-frame of one month.

#### 2.3.2 Baseline-derived risk factors for discrepancy

**Figure 1:**
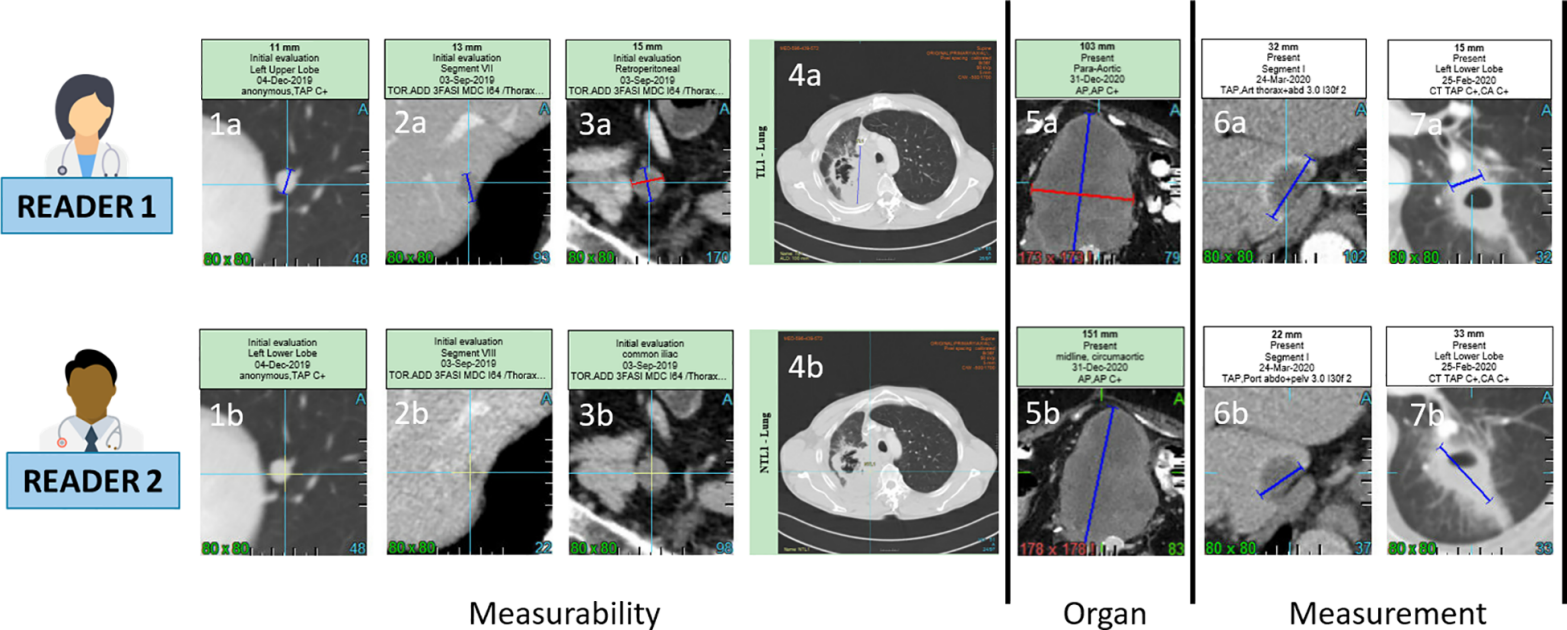
Inter-observer discrepancies at baseline. For a same lesion, two radiologist might consider non measureable a lesion due to its size not meeting the RECIST 1.1 measurability criteria of 1cm long axis for non lymph-node lesions (1a,1b,2a,2b) and 1.5cm short axis for lymph node (3a,3b). The measureability criteria can also be challenged for large lesions with ill defined margins and non robust measurement (4a,4b). Two radiologist might consider one same disease lesion belonging to lymph-node or not lymph-node organ resulting in a large measurement discrepancy due to the different method of measurement for the two type of organs (5a,5b). Measurement discrepancy can also be linked to the selected series for measurement such as arterial versus portal phase (6a,6b) or to the type of lesions such as cavitary lesions (7a,7b).

**Figure 2:**
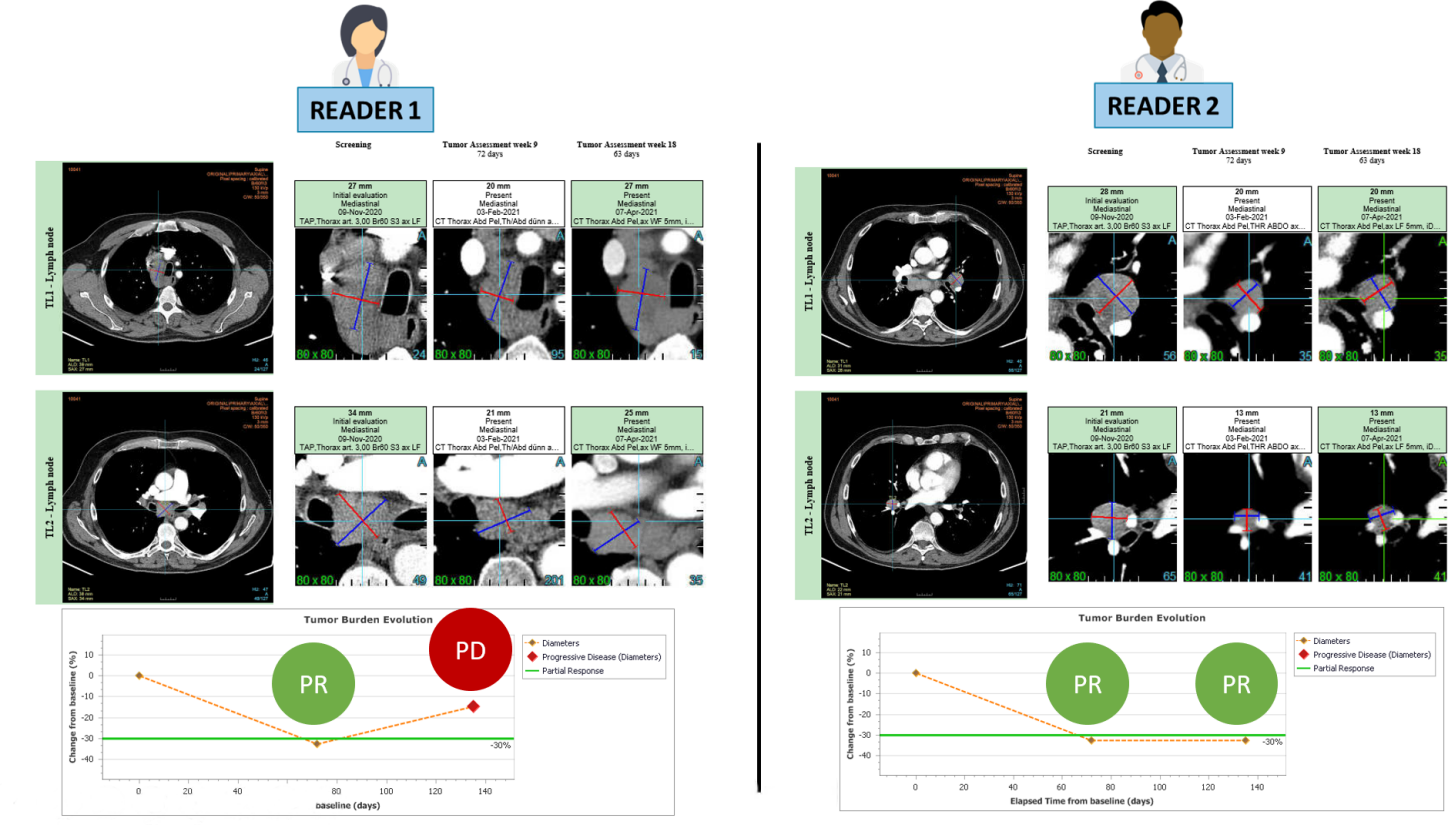
RECIST 1.1 inter-reader variability at baseline leading to endpoint discrepancy. The patient is presented at baseline with involvement of several mediastinal and hilar lymph nodes. Both readers followed RECIST 1.1 guidelines and accurately selected 2 large lymph nodes for measurement without any errors. During the follow-up period, both readers observed a partial response, with the first response documented in week 9. However, at week 18, the readers disagreed on the assessment of disease progression.

We wanted to identify the variabilities in the RECIST process of selection and/or measurement performed at baseline (**Figure 1**) which were likely to entail discrepancy in responses (**Figure 2**). For this aim, we arbitrarily considered risk factors likely 1) to quantify measurement variability at baseline in computing the Delta Burden between the two readers as the relative difference of their SOD (Abs(SOD1-SOD2)/(SOD1+SOD2)) and in measuring SPropSOD (**Annex C**); 2) to quantify the variability in TL selection at baseline in reporting when the two readers did not select their TLs in exactly the same organs, when they selected TLs in totally different organs, or when one of the reader selected a TL in a particularly infrequent location. We also reported when, at least, one of the readers did not select any TL at baseline. At least, we considered a risk factor when the two readers did not select all their NTL in the same organs. Comprehensive description of all the risk factors are provided in **Annex A**. By means of odds ratios (ODDs), we performed a univariate analysis testing associations between KoDs and a set of predefined features (14) (**Annex A**) derived from risk factors. These features applied to target lesions (TLs) and non-target lesions (NTLs) and were stratified according to the different diseased organs (See **Annex B**).

#### 2.3.3 Predictions of discrepancy derived from baseline evaluations

Since the association of a risk factor with a given outcome must be strong (ODD>10) to make classification effective (15), we used previously identified risk factors to train a ML model. After features reduction and classification, we documented the performances of two classification systems.

### 2.4 Statistics

All statistics were performed using base version and packages from R CRAN freeware.

Confidence Intervals (CIs) of discrepancies rates were computed by Clopper-Pearson exact CI method (16). We used “PropCIs” package. Multiple comparisons of continuous variables were performed using the Dunnett-Tukey-Kramer method for unequal sample size (17) with “DTK” package. Multiple comparisons of proportions were performed using Marascuilo test (18).

We derived the proportions of detected KoDs from the 95^th^ percentile of patient follow-up duration in trials and the 95^th^ percentile of follow-up duration until the first occurrence of KoDs. ODDs were computed using “fmsb” package (19), with associated p-values for significant associations.

Continuous variables were analyzed using two samples non-parametric Wilcoxon test (discrepant versus non-discrepant patient groups).

A predictive model of discrepant patient evaluation was trained and tested in a cross-validation with 80:20 split setting. We reported classification accuracy when McNemar’s test indicated no significant bias in assessments due to imbalance in the data. We also reported the Area Under the Curve (AUC).

We evaluated the classification performances using two different algorithms: 1) a random forest (RF) algorithm (20) from the “caret” package after recursive feature elimination (21) and 2) a deep learning (DL) algorithm from the “h2o” package (22) after grid search. CIs were computed for AUC, accuracy (Acc), sensitivity (Se), specificity (Sp), positive predictive value (PPV), and negative predictive value (NPV) using the bootstrap method from the “DescTools” package.

## 3 Results

### 3.1 Discrepancy rates

**Table 3** provides a summary of the KoD rates obtained at the end of each trial. Per KoD, Marascuilo tests yielded no significant inter-trial differences (p>0.05). The average discrepancy rates were 21.0% [19.1; 23.0%], 41.0% [38.7; 43.4%], 28.8 [26.6; 30.9], 48.8% [46.4; 51.2%] for PDD, DOPD, BOR, DOFR, respectively. When combining the data from all five trials, a multiple comparison test showed that there were significant differences between the two KoDs related to time (DOPD and DOFR) and the two KoDs related to the event (PDD and BOR). The discrepancy rate for PDD was lower than for the other KoDs.

**Table 3:** Discrepancy rates at end of trial.

	PDD % (n)	DOPD % (n)	BOR % (n)	DOFR % (n)
**Trial 1** *(N=333)*	16.5 (55)	40.8 (136)	27.0 (90)	45.0 (150)
**Trial 2** *(N=493)*	21.2 (104)	39.3 (194)	26.8 (132)	49.7 (245)
**Trial 3** *(N=243)*	21.4 (52)	33.7 (82)	33.7 (82)	55.6 (135)
**Trial 4** *(N=276)*	26.1 (72)	41.3 (114)	31.9 (88)	51.9 (143)
**Trial 5** *(N=379)*	20.8 (79)	47.7 (181)	27.4 (104)	44.6 (169)
**Pooled** *(N=1724)*	21.0 (362)	41.0 (707)	28.8 (496)	48.8 (842)

For the five clinical trials (rows), we reported, per patient, the double read discrepancy rates as percentages (raw number are in parenthesis). These were computed for each KoD (column): 1) discrepant PDD; 2) discrepant DOPD; 3) discrepant BOR; and 4) discrepant DOFR.

BOR, best overall response; DOFR, date of first response; DOPD, declaration of progressive disease; PDD, progressive disease declared.

**Figure 3:**
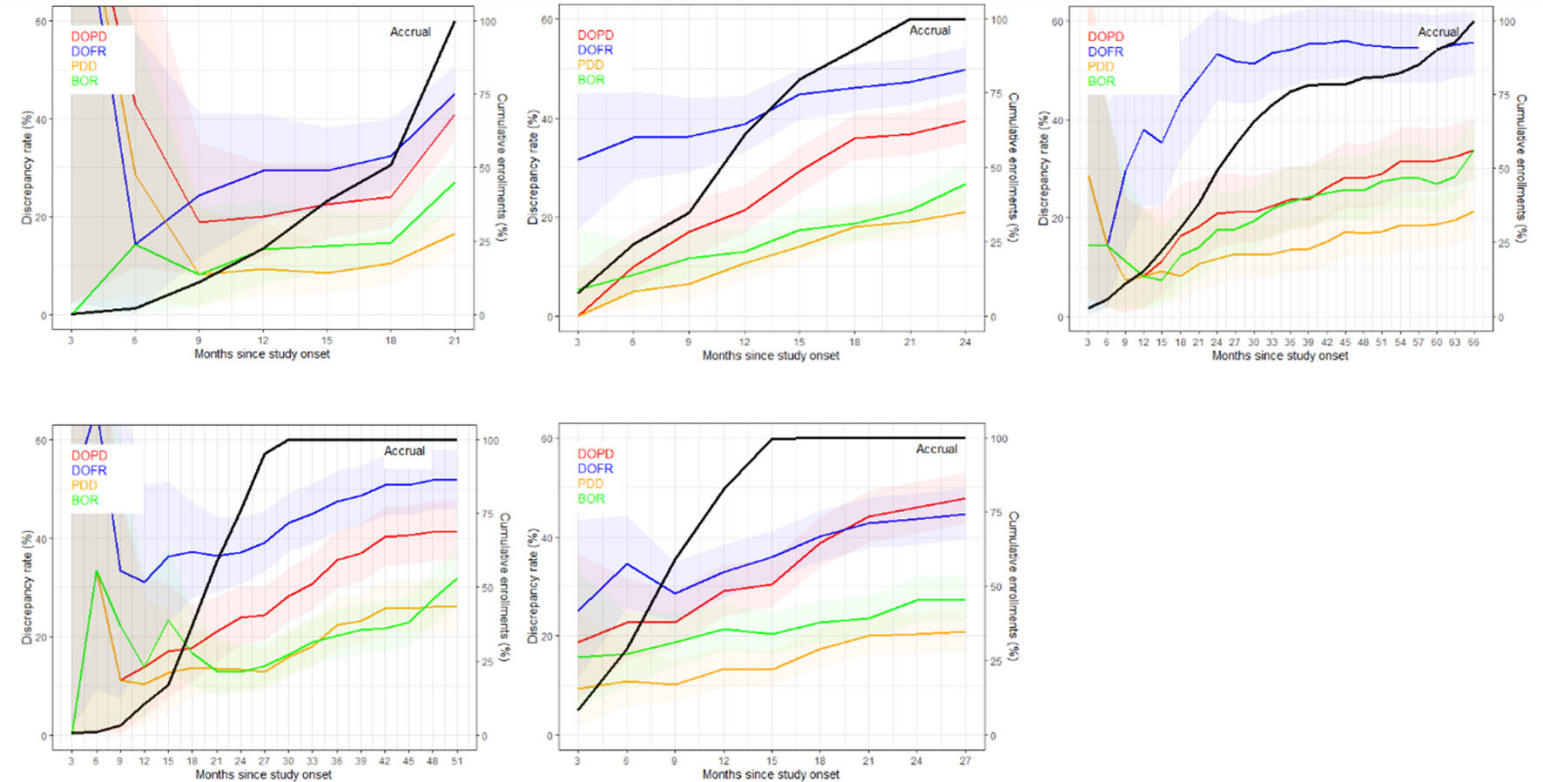
The discrepancy rate over time. The discrepancy rates for the four KoDs are displayed along with the proportion of accrued patients. Curves for DOPD, PDD, DOFR, and BOR KoDs are displayed in red, orange, blue, and green, respectively, with corresponding 95% CIs. The black curve is the cumulative proportion of accrued patients. The five trials are represented from top left to bottom right: a) Trial 1, b) Trial 2, c) Trial 3, d) Trial 4, and e) Trial 5 Time scale is one month.

**Figure 3** displays the discrepancy rates over time for the five trials and the different KoDs, showing that, most of the time, DOFR was higher than DOPD. We observed that the rates of all KoDs increased as the trial progressed, even long after the completion of patient accrual. The KoD curves did not always feature smooth variations, and the curves of accrual displayed different shapes. It seems that a significant patient recruitment immediately from the start of the study will guarantee early meaningful KoD curves.

**Figure 4:**
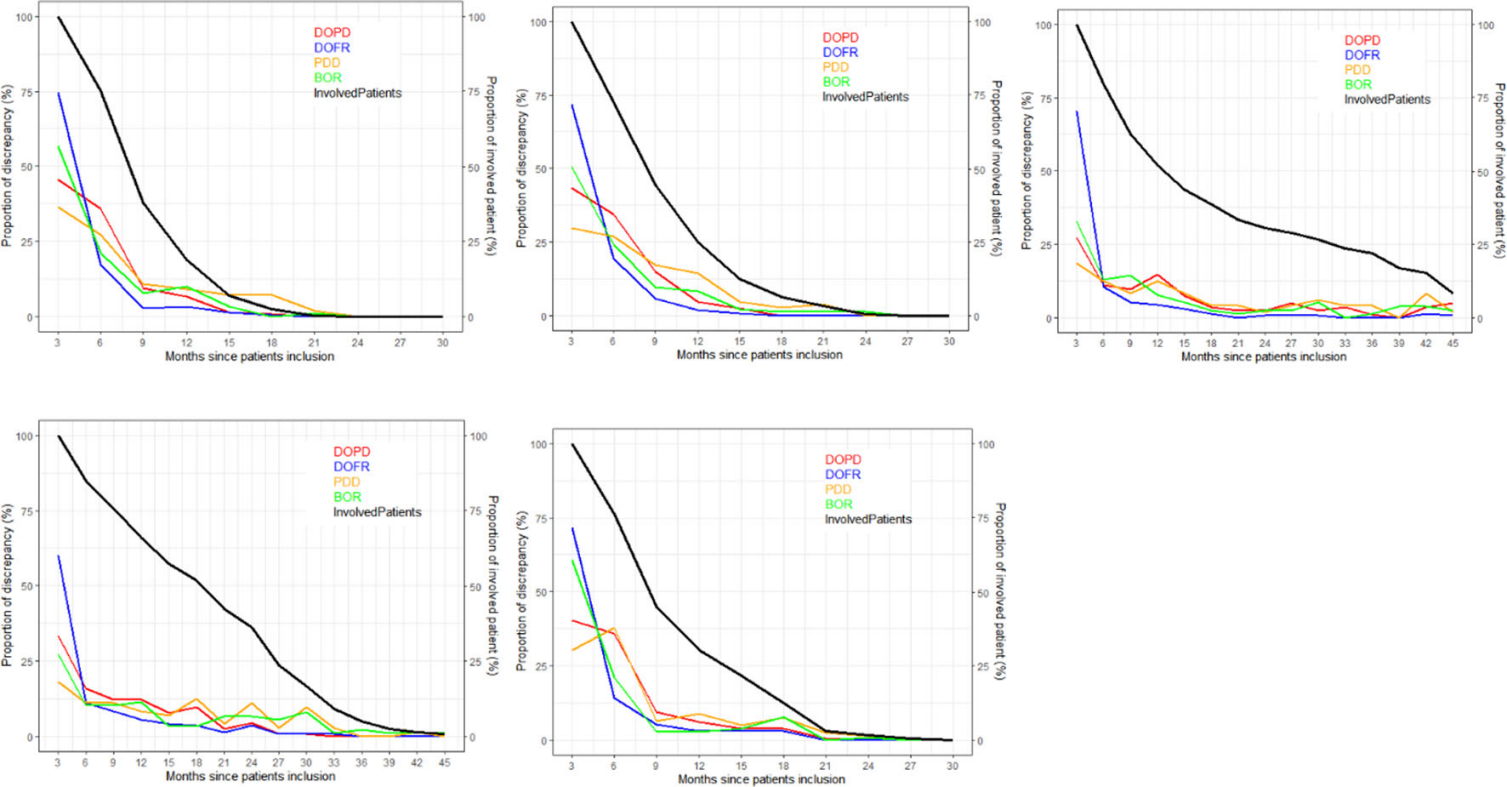
Proportion of discrepancies as distributed during follow-ups. For each trial and KoD, we computed the proportion of discrepancies (Equation 3) occurring at each follow-up. The DOPD, PDD, DOFR, and BOR KoDs are displayed in red, orange, blue, and green, respectively. The black curve displays the proportion of patients evaluated at this time point. BOR, best overall response; DOFR, date of first response; DOPD, declaration of progressive disease; KoD, kind of inter-reader discrepancy; PDD, progressive disease declared.

As depicted in **Figure 4**, the ratio of KoD occurrence and the proportion of patients remaining at this time point followed a steady downward trend over time. At the outset of follow-up, proportionally more DOFR and BOR occurred than DOPD and PDD. Additionally, **Figure 4** demonstrates that the decrease in the proportion of KoD occurrence had distinct patterns. For some trials (Trials 1, 2, and 5), the decrease had a tendency similar to the proportion of patients still present at this time, while for others, this was not the case (Trials 3 and 4).

**Figure 5:**
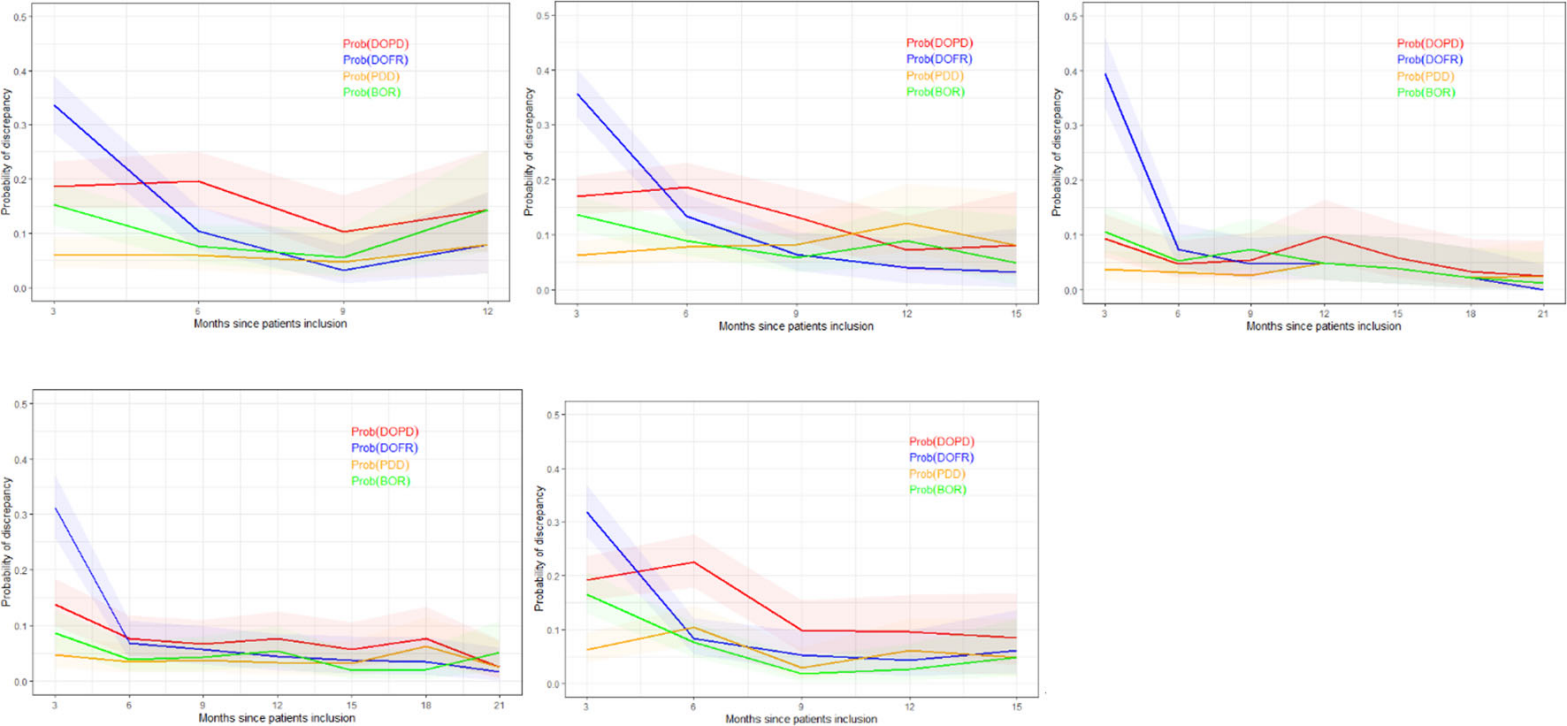
Probability of discrepancy over patients follow-up. The probability of discrepancy for the four KoDs is displayed. Probabilities of DOPD, PDD, DOFR, and BOR KoD occurrence are displayed in red, orange, blue, and green, respectively, with corresponding 95% CIs. The five trials are represented from top left to bottom right: a) Trial 1, b) Trial 2, c) Trial 3, d) Trial 4, and e) Trial 5. BOR, best overall response; CI, confidence interval; DOFR, date of first response; DOPD, declaration of progressive disease; KoD, kind of inter-reader discrepancy; PDD, progressive disease declared.

In **Figure 5**, we present the probability of KoDs occurring in relation to consecutive time points.

In **Figure 5**, we can see that the probability of discrepancies occurring with DOFR was significantly higher at the beginning of follow-up, but became closer to the other KoDs at the following time point.

After 6 months, the probabilities of Trial 3 and 4 KoDs were generally less than 10%. They were higher for other trials. DOFR was the most likely KoD in the initial part of the patient evaluation, and the ordering of KoDs was stable when considering phase 3 studies i.e., DOFR > DOPD > BOR > PDD.

Regarding progression-related KoDs, the probability of PDD at the patient level was quite stable over cycles while the probability of DOPD tended to decrease over cycles. PDD was also globally lower than the KoDs of response at the beginning of evaluations.

### 3.2 Evaluation of risks factors derived from baseline discrepancies

We found that for a single trial (Trial 3), one reader did not identify any disease in two patients out of 243 (0.8%), without any evidence that there was a significant risk factor of discrepancy, particularly for DOPD (p=0.68) or DOFR (p=0.1).

Except for Trial 3, classification of disease as non-measurable by at least one reader occurred in less than 7% of patients. It was very common for readers to select some of their TLs and NTLs in different organs, ranging from 36.0-57.9% and 60.5-73.5% of patients, respectively, but rarely (4.0-8.1%) did the two readers select all of their TLs in different locations.

We did not identify any set of risk factors which was relevant to all trials for a given KoD.

As summarized in **Table 4**, significant risk factors varied depending on the KoD and the trial examined. Analysis of pooled data revealed significant risk factors associated with BOR, whereas DOPD had a single risk factor (non-measurable disease reported by one reader) that could be identified at baseline. PDD had none. One or more readers choosing an infrequent disease location was not a risk factor for discrepancy.

**Table 4:** Risks factors and occurrence of discrepancy.

	Non-measurable disease (NTLs)	TLs not all in same organs	All TLs in different organs	NTLs not all in same organs	Delta burden	TLs not lung for one of the readers	SPropSOD*	Infrequent disease location
**Trial 1** **(N=333)**	BOR (4.8%)	BOR (42.9%)	(7.6%)	BOR PDD (61.5%)	BOR (NA)	BOR DOFR (23.3%)	PDD (NA)	(6.6 %)
**Trial 2** **(N=493)**	(0.4%)	(40.9%)	BOR DOFR (6.3%)	PDD (66.9%)	BOR DOFR (NA)	BOR DOFR (13.0%)	BOR DOFR DOPD (NA)	DOFR (7.9 %)
**Trial 3** **(N=243)**	BOR PDD (22.9%)	(47.0%)	BOR (8.1%)	DOPD (63.7%)	(NA)	(13.1%)	(NA)	BOR DOFR (17.1%)
**Trial 4** **(N=276)**	BOR DOFR (6.1%)	(57.9%)	DOFR DOPD (5.8%)	(73.5%)	DOPD (NA)	BOR DOPD (25.1%)	BOR (NA)	(43.1 %)
**Trial 5** **(N=379) **	BOR (1.3%)	(36.0%)	(4%)	(60.5%)	DOFR (NA)	(13.1%)	PDD (NA)	(9.0 %)
**Pooled (N=1724)**	BOR (4.7) DOFR (1.7) DOPD (0.6) (5.5%) 95/1718	BOR (1.3) (43.5%) 706/1623	BOR (1.8) DOFR (1.9) (6.2%) 100/1623	DOFR (1.3) (65.1%) 1120/1720	BOR (1.7) DOFR (1.6) (NA)	BOR (1.7) DOFR (1.4) (17.0%) 276/1623	BOR (1.8) (NA)	(14.8%) 255/1720

For the five clinical trials (rows) and the different risk factors (columns), we reported the KoDs representing a significant risk: DOPD (red), PDD (Orange), DOFR (blue), BOR (green). In parentheses of corresponding colors are the values of the ODDs. In black parentheses are the percentages of patients with the potential risk factors. ODDs derived from Delta Burden and SPropSOD (Annex C) used optimized thresholds, so represented best cases.

BOR, best overall response; DOFR, date of first response; DOPD, declaration of progressive disease; NTL, non-target lesion; PDD, progressive disease declared; SPropSOD, percentage of specific sum of diameters; TL, target lesion.

### 3.3 Prediction of discrepancy derived from baseline evaluations

Our risk factors analysis showed that progression-related KoDs were marginally impacted by baseline evaluation. Therefore, our evaluation of predictive models focused mainly on the response-related KoDs of DOFR and BOR.

Based on our features set (14), and a pre-processing of feature selection for RF algorithm, classification performances for response-related KoDs are summarized in **Table 5**. For each independent clinical trial or in pooled data, feature selection did not improve classification performances.

**Table 5:** Prediction of response KoDs derived from baseline features.

	DOFR	BOR
**AUC**	57.2[56.6; 57.7]	60.8 [60.2; 61.4]
**Acc**	55.4 [54.9, 55.8]	73.1 [72.8, 73.3]
**1. Se** **2. Sp** **3. PPV 4. NPV**	44.1 (43.1; 45.1) 66.2 [65.3; 67.2] 56.0 [55.3; 56.2] 55.5 [55.0; 56.0]	12.4 (11.8; 12.9] 97.4 [97.1; 97.6] 66.0 [63.0; 68.0] 73.5 [73.0; 74.0]
**AUC**	58.0 [57.7; 58.2]	61.9 [61.6; 62.2]
**Acc**	52.8 [52.3; 53.3]	73.5 [73.0; 73.9]
**1. Se** **2. Sp** **3. PPV** **4. NPV**	4.3 [3.5; 5.0] 98.9 [98.6; 99.2] 84.0 [81.6; 86.4] 52.2 [51.6; 52.7]	10.3 [9.4; 11.2] 98.8 [98.6; 99.0] 81.0 [78.8; 83.2] 73.3 [72.8; 73.8]

We pooled the data from the five trials to evaluate classification performance of two algorithms in a cross-validation setting: RF after feature selection (Top rows, in blue); DL after grid search of the hyperparameters (bottom rows, in green). For the KoDs of response, we measured the predictive performances as the AUC, Acc, Se, Sp, PPV, and NPV with corresponding CIs (in block brackets).

Acc, accuracy; AUC, area under ROC curve; BOR, best overall response; CI, confidence interval; DL, deep learning; DOFR, date of first response; KoD, kind of inter-reader discrepancy; NPV, negative predictive value; PPV, positive predictive value; RF, random forest; Se, sensitivity; Sp, specificity.

Using the validation dataset of pooled data, DL outperformed the RF algorithm. Performances with DL were poor but with a PPV higher than 80% for all the KoDs. The best classification performances were obtained with BOR based on AUC. For DOPD, AUCs was 57.3 [57.1; 57.5].

## 4 Discussion

### 4.1 Discussion of our results

At completion of trials, discrepancy rates based on DOPD or DOFR were comparable across trials with respective average values of 41.0% and 48.8%. Over time, the rates of KoDs steadily increased as the trials progressed, even after the end of patient accrual. The discrepancy rates for the time-related endpoints, DOPD and DOFR, were always higher than their event-related PDD and BOR counterparts. A higher proportion for DOPD was expected as the counting of DOPD occurrences encompassed those of PDD. Translated into clinical study endpoints, these observations mean that discrepancy rates for overall response rate are generally higher than for PFS.

Assuming part of the discrepancies was attributable to a delayed event detection by one of the readers, it could be expected that the proportion of DOFR would be higher than DOPD at an earlier stage, as progressive patients were withdrawn from the trial; the only room for patient response was then before progression.

As **Figures 3**–**5** show, the number of recruited patients, *PatIncl(t)*, at each time point is an operational-related function that can significantly vary between trials and is difficult to predict. The survival curve, *Surv(t*) (and the PFS curve), however, is dependent on the drug and/or disease, and can be predicted to some degree. The likelihood of a discrepancy occurring at each time point is more of a measure of the reading process, which is partially dependent on the readers’ abilities and the complexity of the observed disease. To predict the rate of discrepancies over time, one must be able to completely regulate the trials, the drugs, and the readers’ performances. The mathematical formulation of the temporal discrepancy rate (Equation 1) helps us understand the intricacies of trials by breaking down the components that interact with one another. When the rate of discrepancies increases, it can be difficult to determine if the cause is a pattern of patient recruitment or if readers are simply more prone to making mistakes.

Regarding our risk factor analysis, we found that at least one reader not detecting disease when it was likely present was very rare and not a major concern. Selecting non-measurable disease (i.e., NTLs only) was more prevalent and was considered a significant risk when pooling the five trials.

The analysis of discrepancies between DOPD and DOFR showed that readers often selected TLs (43.5% of patients) or NTLs (65.3%) in different organs without being critical of discrepancies. When all the data were pooled, most of the risk factors were significant for BOR. Selecting non-measurable disease was the only risk factor for DOPD discrepancies. The selection of infrequent disease by any reader was not a risk factor regardless of the KoD.

Feature selection did not improve RF classification performance. DL slightly outperformed the RF algorithm but with globally poor classification performance. Best performances (AUC based) were reached in detecting BOR (61.9).

### 4.2 Discussion around the literature

#### 4.2.1 Discrepancy rate

The discrepancy rates we found at end of trial agree with the literature (8, 23). Considering the naïve assumption of equiprobability between the four RECIST classes of response, the ranking of our measured discrepancies rates DR(DOFR) > DR(DOPD) are consistent with the basic rules of combinatory probabilities.

#### 4.2.2 Discrepancy timing

Most discrepancies occurring earlier for DOFR than for DOPD can be explained by the fact that a genuine response can only take place before a progression (except for pseudo progressions), because patients are withdrawn from trials after a PD.

As we observed in **Figure 2**, the ranking of KoD rates could change over time (crossing curves). This indicates that the probability of inter-reader discrepancy is not a stationary process in time (24). This factor should be considered when it comes to improving the modeling of trial monitoring, as illustrated by Equation 1, and when designing new metrics. The probability of PDD at each cycle seemed the most stable and lower than other KoDs.

#### 4.2.3 Risk factors

##### 4.2.3.1 NTL assessment

The NTL category only determines three events: CR, PD, and stability (Not CR/Not PD). The PR event is only determined by measurable disease. In support of this finding, Raskin et al. (25) showed that NTLs were an important factor for detecting PD, while Park et al. (26) revealed that selecting metastasis-only lesions as TLs may be more effective for determining response in kidney disease.

In our observations, for more than 5% of patients, at least one reader did not detect any measurable disease, so only NTLs were selected. Under these conditions, it is not surprising to observe a significant correlation with the occurrence of a response-related KoD (**Table 4**).

Moreover, the NTL category theoretically includes less defined and smaller lesions, making them more equivocal during the first evaluation of the disease. The high prevalence of selected NTLs in different organs (65% of patients) reflects this uncertainty when capturing the disease at baseline. Lheureux et al. (27) developed a comprehensive discussion about the equivocality associated with RECIST, which is responsible for concerns related to its reliability. Moreover, during follow-up, due to the “under-representation” of this category and the qualitative appreciation of non-measurable disease, RECIST recommends interpreting progression of NTLs by considering the entire disease. Indeed, it is rare to observe a PD event triggered solely by the NTL category. This is reported during paradoxical progression in approximately 10% of progression cases (28). Finally, since the CR event is quite rare in our patients in advanced clinical stage, the influence of a difference in the appreciation of the non-measurable disease ultimately presents little risk in terms of variability on the study’s endpoints. Cases that are ultimately more at risk relate to patients with a low measurable tumor mass compared to the non-measurable disease. However, the detection of these cases is very difficult in the absence of quantification of NTLs [see scenario F in the supplementary appendix of Seymour et al. (29)].

##### 4.2.3.2 TL selection

We showed that readers selected TLs in different organs in 36.0% to 57.9% of patients, with no association with DOPD or DOFR discrepancies and poor association with BOR.

In the study by Keil et al. (12), for 39% of patients, readers had chosen different TLs, demonstrating a strong association with DOPD. Keil et al. had different study inclusion criteria, considering breast cancer, a single follow-up, no new target and no NTLs. Their mean number of TLs was 1.8 (2.3 in our study). Keil et al. adopted a strict definition of “same TLs” as those with the same coordinates, whereas ours was for those chosen in the same organ.

Kuhl et al. (30) reported higher discrepancy rates than us (27% for DOPD). Readers selected different sets of TLs in 60% of patients with even a stronger association with readers’ disagreement than Keil et al. Kuhl et al. adopted an even stricter definition of concordant selection than Keil et al. Kuhl et al. also included a broad spectrum of primary cancers.

##### 4.2.3.3 Sum of diameters value

Our study confirmed findings by Sharma et al. (31) who concluded that there was an association of the variability of SOD at baseline with the variability of the study endpoint. However, our percentage of specific sum of diameters (SPropSOD) analysis did not confirm or contradict other works about dissociated responses (28, 32), probably because this phenomenon is reportedly observed in only around 10% of cases (28).

##### 4.2.3.4 Location (TL lung & infrequent)

Regarding discrepancies in targeting the most frequent location, this was poorly associated with variability of responses. We considered five primary lung cancer trials, but for 17% of the patients, at least one reader did not select any lung TL.

Regarding discrepancies in identifying disease (TL or NTL) in infrequent locations, surprisingly we found no risk factors associated, although some authors discuss the controversial use of RECIST outside the most targeted disease locations (33).

##### 4.2.3.5 Progression-related KoDs

Several studies (34, 35) have documented that more than half of discrepancies in reporting progressing disease are triggered by debatable detection of new lesions. Thus, they are not concerned with baseline evaluations, preventing prediction from baseline. Unlike Keil et al. (12), we did not measure a systematic risk factor associated with TL selection. The only exception was in a specific trial (Trial 4), when readers selected all of their TLs from completely different organs.

#### 4.3.3 Classifications

According to Corso et al. (20), feature selection does not improve classification performances. For all the KoDs, DL outperformed RF. Overall, classification performance was low. Specifically regarding detection of DOPD, our findings were consistent with studies that reported that the majority of DOPD discrepancies were due to the misdetection of new lesions (34), which is not linked to baseline. The best classification performances were obtained for BOR, albeit with poor performances.

Even though certain studies suggest that baseline selection and measurement have considerable influence on the accuracy of response assessment (12, 13), we found that, while they existed, these correlations were weak and heterogeneous. Therefore, additional root causes of variability could be studied, such as follow-up management (e.g., measurement variability of tumor burden or perception of NTL change), readers’ associations, or fluctuating readers’ perception (35). We also need to investigate the gap between selecting “exactly the same TLs” and “TLs in same organs”, as the first definition is reported to have a strong association with variability in literature (36), while we found the second has a weak association. If strong heterogeneity within the same organ is confirmed, the issue of RECIST is no longer its subjectivity but its intrinsic inappropriateness in assessing patient follow-up.

#### 4.3.4 Study limitations

First, we did not document some well-known risk factors for variability linked to image quality, such as variability due to reconstruction parameters (different image selection) or the timing in IV contrast injection. Indeed, we assumed that the imaging charter of the included trial adequately standardized the images so that these risks associated with these factors would be negligible.

Second, we did not analyze the impact of the selection of the same TLs by readers. When designing our study, we did not expect that collecting tumor coordinates would be of interest but assumed that checking the association with TLs in the same organ would be adequate.

Third, we did not investigate the impact of the inter-reader variability in assessing the “measurability” of tumor. Indeed, when a first reader considers a tumor as measurable and candidate to be TL included in his tumor burden, while the second reader considers the same tumor as NTL, the two readers would have a different in sensitivity at detecting responses.

Fourth, our study focused on lung data, therefore cannot be generalized to other diseases. For some other types of cancers CT is not the preferred modality, the disease spread differently, and the tumors feature different phenotypes, thus risk factors and training performances would be different.

Lastly, as we were blinded as to randomization, we were not able to refine our analysis by treatment/control. However, we can assume that KoD statistics and association with variability is different for treatment and control as those statistics are directly linked to the occurrence of the events of response or progression, supposedly different for the two arms.

#### 4.3.5 Perspective on operations

Throughout the initial cycles of BICR with double reads, the rate of discrepancies is hard to analyze without a benchmark to refer to. This leads researchers to search for other key performance indicators to assess trial reliability and, eventually, take corrective measures as expeditiously as possible.

Even though we were not able to make powerful predictions, our analysis of the baseline data revealed that some inter-reader differences can affect the reliability of trial results. A tracking of baseline assessment during BICR could be a beneficial addition to trial quality control.

The poor predictive performances were probably, in part, obtained because a predictive model cannot avoid including follow up data. Another hypothesis could be that, based only on baseline data, the risk factors, and the features we considered were unable to fully capture the complexity of disagreements. In the future, we can imagine improving the features derived from tumor selection or even creating new ones; considering other risk factors in the classification, such as variability in scan selection or variabilities in involving the first follow-up and optimizing readers’ associations. However, an open question will remain: “How can we manage a baseline assessment with a high probability of becoming discrepant?”. If future technologies can predict discrepancies, it is likely to be a prediction with limited explicability. Moreover, we can question whether the 2 + 1 adjudication paradigm is obsolete, as choosing between two medically justifiable differences (2) does not make sense.

## 5 Conclusion

For the discordance rate to be predictable over time, at each time point, we need to know patient accrual, patient survival, and probability of discrepancy. Discrepancies in date of responses occurred more often than those related to progressions. Careful thought should be given to corrective actions based on the analysis of KoD rates if less than 50% of patients have been enrolled.

Several risk factors for inter-reader discrepancies have been confirmed, albeit with relatively weak implications. We found that for around 50% of patients, readers chose tumors in different organs without impacting the variability of responses. The prediction performances of inter-reader discrepancies based on the baseline selection were poor. Baseline-derived features should be improved or new ones designed, other risks factors must be considered for predicting discordances, and optimal reader association must be investigated.

## 6 Take home messages

1) The discrepancy rate over time depends on patient accrual, PFS, and the probability of discrepancy during follow-up.
2) At the outset of follow-up, proportionally more DOFR and BOR occurs than DOPD and PDD.
3) The KoD rates are not stabilized in the first half period of total patient inclusion and should be interpreted carefully. Baseline variability evaluation can help to determine risk of study endpoint variability.
4) The inter-reader variability in disease selection at baseline is frequent (50%). The general impact on the variability is more significant for response endpoints.

## Data availability statement

The data analyzed in this study is subject to the following licenses/restrictions: Data set are proprietary to sponsors. Requests to access these datasets should be directed to https://clinicaltrials.gov.

## Author contributions

HB: Conceptualization, methodology, data curation, formal analysis, original draft, writing, review, and editing. AI: Conceptualization, methodology, formal analysis, project administration, original draft, review, and editing. Both authors contributed to the article and approved the submitted version.

## Annexes

### A. List of features

**Table:**

ID#	Feature abbreviation	Feature description
1	SumTL	Sum of the number of TLs selected by the two readers.
2	MinTL	Minimum number of TLs selected by the readers.
3	DeltaTL	Difference in the number of TLs selected by the two readers.
4	DeltaBurden	Relative difference of SOD between the two readers: Abs(SOD1-SOD2)/(SOD1+SOD2).
5	MeanBurden	Average SOD measured by the two readers.
6	NoDisease	No disease was found by at least one reader.
7	NonMeasurable	Non-measurable disease (TL) was found by at least one reader.
8-39	TL Organ	One of the readers selected their list of TLs in given organs from the catalogue (See Annex B), while the other reader did not use the same list.
40	NotSameLoc	Binary: At least one TL selected in different organs by readers.
41	NoIdenticalLoc.	Binary: Readers did not select any TL in common organ.
42-73	NTL Organ	One of the readers selected their list of NTLs in given organs from the catalogue (See Annex B), while the other reader did not use the same list.
74	NotSameNTLLoc	Binary: At least one NTL selected in different organs by readers.
75	NoIdenticalNTLLoc.	Binary: Readers did not select any NTL in common organ.
76	SPropSOD	% of specific SOD. (See Annex C)
77	Unfreq. Disease	Binary: At least one reader selected an infrequent disease [#10; #31].

NTL: non-target lesion; SOD: sum of diameters; TL: target lesion.

### B. List of disease locations

**Table:**

1. Lung	2. Liver	3. Lymph node	4. Pleura	5. Chest wall
6. Bone	7. Abdominal wall	8. Adrenal gland	9. Brain	10. Spleen
11. Bone marrow	12. Esophagus	13. Kidney	14. Mediastinum	15. Peritoneum
16. Muscle	17. Subcutis	18. Pericardial cavity	19. Skin	20. Pancreas
21. Gastric	22. Blood vessels	23. Heart	24. Neck	25. Spinal cord
26. Thyroid	25. Diaphragm	28. Pelvis	29. Breast	30. Trachea

• additional category, the 31st, labeled as “miscellaneous” was added. • Infrequent disease locations are those from 10 (spleen) to 31 (miscellaneous).

### C. Specific proportional sum of diameter

Considering:

SODi: Tumor burden as reported by reader i

C_SOD_i_: Part of the tumor burden that targets same organs selected by the other reader

S_SOD_i_: Part of the tumor burden that targets organs not selected by other reader $SOD_{i}=C\_SOD_{i}+S\_SOD_{i}$ Equation 1

The specific SOD can be defined as: $SPropSOD=100*S\_SOD_{i}/SOD_{i}$ Equation 2

So that, when a reader targeted the same organs SPropSOD=0 while when readers targeted completely different organs SPropSOD= 1
